# Assessment of regional air quality by a concentration-dependent Pollution Permeation Index

**DOI:** 10.1038/srep34891

**Published:** 2016-10-12

**Authors:** Chun-Sheng Liang, Huan Liu, Ke-Bin He, Yong-Liang Ma

**Affiliations:** 1 State Key Joint Laboratory of Environment Simulation and Pollution Control, School of Environment, Tsinghua University, Beijing 100084, China; 2 State Environmental Protection Key Laboratory of Sources and Control of Air Pollution Complex, Tsinghua University, Beijing 100084, China; 3 Institute of Atmospheric Environment, Chinese Research Academy of Environmental Sciences, Beijing 100012, China

## Abstract

Although air quality monitoring networks have been greatly improved, interpreting their expanding data in both simple and efficient ways remains challenging. Therefore, needed are new analytical methods. We developed such a method based on the comparison of pollutant concentrations between target and circum areas (circum comparison for short), and tested its applications by assessing the air pollution in Jing-Jin-Ji, Yangtze River Delta, Pearl River Delta and Cheng-Yu, China during 2015. We found the circum comparison can instantly judge whether a city is a pollution permeation donor or a pollution permeation receptor by a Pollution Permeation Index (PPI). Furthermore, a PPI-related estimated concentration (original concentration plus halved average concentration difference) can be used to identify some overestimations and underestimations. Besides, it can help explain pollution process (e.g., Beijing’s PM_2.5_ maybe largely promoted by non-local SO_2_) though not aiming at it. Moreover, it is applicable to any region, easy-to-handle, and able to boost more new analytical methods. These advantages, despite its disadvantages in considering the whole process jointly influenced by complex physical and chemical factors, demonstrate that the PPI based circum comparison can be efficiently used in assessing air pollution by yielding instructive results, without the absolute need for complex operations.

Air pollution is primarily measured by air quality monitoring networks (AQMNs) in many countries. AQMNs characterize pollution patterns and determines compliance of pollutants with air quality standards. Thus, it protects the public health^1^,^2^,^3^. Numerous modeling methods have been found in literature to assess the characteristics, processes, sources and impacts of air pollution. Some of the methods include transport and dispersion simulations (source models), such as Hybrid Single-Particle Lagrangian Integrated Trajectory (HYSPLIT)^4^,^5^,^6^,^7^, Community Multiscale Air Quality (CMAQ)^8^,^9^,^10^,^11^,^12^,^13^, and potential source contribution function (PSCF)^6^,^7^,^14^. Many receptor models, such as positive matrix factorization (PMF)^15^,^16^,^17^,^18^,^19^,^20^,^21^, principal component analysis with multiple linear regression analysis (PCA-MLRA)^6^,^22^,^23^, and chemical mass balance (CMB)^24^,^25^ are also widely used. Besides, there are many other numerical modeling and analytical methods such as GEOS-Chem chemical transport model^12^,^24^,^25^,^26^,^27^,^28^,^29^,^30^,^31^,^32^,^33^,^34^, Weather Research and Forecasting model coupled with chemistry (WRF/Chem)^29^,^35^,^36^,^37^, land-use regression (LUR) models^38^,^39^,^40^,^41^, Dust REgional Atmospheric Model^42^, observation-based model^43^, GIS-based multi-source and multi-box modeling^44^, global sensitivity analysis method^45^, and episode-based evolution pattern analysis^46^. In addition to using such methods to characterize the amount of pollution level in selected areas, the enhanced air quality index (AQI) can also be used^47^,^48^,^49^, such as in the form of pollutant concentration values divided by their standards^50^. The air pollution monitoring networks have been greatly improved worldwide in recent years with increased and updated sites^51^,^52^. The cities are more connected in a net, which offers better chances to assess regional air pollution.

However, first, the above-mentioned methods are mostly sector related analyses. Only few of them are region related analyses. Second, the region related analyses rely on large amount of data (such as emission inventory, meteorology, mechanism), to form white box models. Third, all the air quality models are calibrated by observational data, namely using the observed results to improve the understanding of the pollution process (such as atmospheric chemistry)^53^, which could be complemented by grey box models established by using direct observation. Fourth, interpreting the monitoring data in simple and efficient way is still a challenge, despite using above-mentioned methods. In other words, there is a need for new analytical methods to assess air pollution using air quality monitoring data to improve the understanding and control measures of regional air pollution. This paper reports such a method whose peculiarities include: (1) region related; (2) requiring much less data; (3) directly using monitoring results, and (4) simple but efficient. This approach is based on the comparison of pollutant concentrations between target and “circum” areas (referred as circum comparison in short), namely a Pollution Permeation Index (PPI) that is concentration-dependent. Circum areas (cities) are the surrounding areas (cities) of a target area (city). PPI is a relative magnitude of concentration difference. It is the potential of an area (city) to permeate pollution to surrounding areas (cities) or to be permeated by pollution of surrounding areas (cities). To ensure this method’s rationalization and standardization for practical use, the influences among areas (cities) discussed in this work are all derived from concentration-difference permeation unless otherwise specified. The proposed method’s applications are tested by assessing the air pollution of four important Chinese regions, namely Jing-Jin-Ji (Beijing, Tianjin, and Hebei), Yangtze River Delta (here means Shanghai, Jiangsu, and Zhejiang), Pearl River Delta (Guangdong) and Cheng-Yu (Chengdu and Chongqing) during 2015, using backward deduction.

The circum comparison approach supports to instantly judge whether a city is a pollution permeation donor or a pollution permeation receptor with Pollution Permeation Index (PPI). Areas with positive PPI values correspond to pollution permeation donors and vice versa. Donor is underestimated or loosened because of its net polluting role and receptor is overestimated or wronged because of its net being-polluted role. Furthermore, PPI provides a kind of estimated concentration for a target city based on its original concentration plus halved average concentration difference. Thus, the overestimations and underestimations of self-made pollution severity can be identified from the pollution-permeation point of view. The donors that are underestimated or loosened should be more strictly controlled for air pollution control, and vice versa. Hence, the PPI can provide quantitative information of interaction among target and circum areas and provide instructions on how to control regional air pollution. This complements the AQI or air pollution index (API) that provides information to inform the public about air pollution^54^. Besides, it can help explain pollution process (e.g., PM_2.5_ in Beijing may be largely promoted by non-local SO_2_) though not aiming at characterizing pollution process. Moreover, it is applicable to other monitored regions and easy for users to quickly get started with it. Thus, it fully broadens the application scope and improve the usage efficiency of existing pollution data. Other new applications and efficient air pollution analytical methods are expected to be developed with PPI that would be foundation of other new analytical methods. These features demonstrate that simple calculation methods can also be used in assessing air pollution, without the absolute need for complex operations such as developing and using models. With better spatial representativeness, accuracy and reliability of monitoring network^51^,^52^,^55^,^56^,^57^,^58^, and future micro-stations using technologies such as durable and solar-powered air monitoring park bench^59^, low-cost micro-scale sensors^60^, the PPI based method may become more reliable and useful.

## Materials and Methods

China has a strong national monitoring network with more than 1400 nation-owned sites (non-state-owned sites are excluded) across its territories^61^. Similarly, for example, the US has 1500 sites maintained by both state-owned and non-state-owned agencies^62^. Air pollution data from air quality monitoring stations under study were taken from http://www.aqistudy.cn/historydata/. We used these data for calculating the Pollution Permeation Index (PPI) in target regions and cities. We selected four well-known regions in China as an example to examine the feasibility of assessing their air quality according to the PPI based methods. These four regions are Jing-Jin-Ji (Beijing, Tianjin, and Hebei), Yangtze River Delta, Pearl River Delta and Cheng-Yu (Chengdu and Chongqing). Some surrounding cities of Chongqing are excluded because of the unavailability of monitoring data. We mainly used the yearly data (annual average) that are calculated from monthly data (Table S1). The approach is limited for yearly cases as only yearly air pollution data of the exampled cities and their neighbor cities was utilized.

### Theoretical Basis and Calculations

A certain city and its surrounding cities were defined as a target city and circum cities respectively for the circum comparison in the study. The specific examples used in this study are shown in Table S2.

PPI is the sum of the concentration difference of pollutants between an area and its surrounding areas divided by its pollutant concentration, a permeation potential by concentration difference, namely the potential of an area to permeate pollution or to be permeated pollution by its surrounding areas purely from the concentration-difference point of view. Mathematically, PPI is represented as I^PP^, and 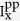 is the PPI of a certain pollutant. For convenience, in short PPI can be called I.


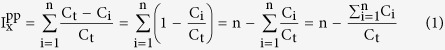


**Figure 1:**
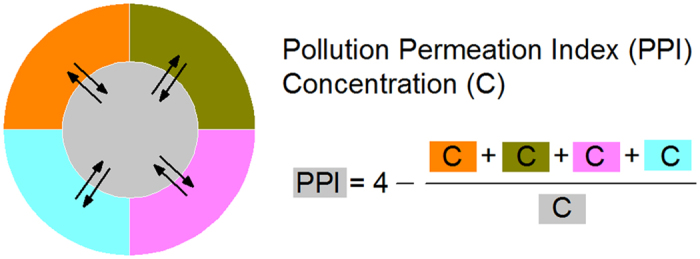
A schematic of an example city (or area) with 4 neighbor cities by the circum comparison method.

In Equation 1, x = PM_2.5_, PM_10_, SO_2_, NO_2_, O_3_, or CO; i is the i-th connected city; and n means the total number of circum cities. Figure 1 shows the overview on the concept.

For a more specific example, Beijing is the target city which has 6 surrounding (circum) cities, namely Zhangjiakou, Chengde, Tangshan, Tianjin, Langfang, and Baoding. The PM_2.5_ PPI of Beijing is 6 minus the sum of PM_2.5_ concentrations of Zhangjiakou, Chengde, Tangshan, Tianjin, Langfang, and Baoding divided by the PM_2.5_ concentration of Beijing. Similarly, each PPI of the other pollutants such as PM_10_, SO_2_, NO_2_, O_3_, and CO of an area can be calculated. We used administrative areas to classify the regions. So, the individual cities are examples of target and comparison objectives in this study. PPI users can classify a region into some (e.g., four equal as Fig. 1 shows) areas according to its geography and meteorology. The black arrows represents the mutual pollution permeation between neighboring cities that dynamically interacts with each other.

Equation 2 represents the mathematical definition of a kind of estimated concentration for an objective area based on its original concentration and halved average concentration difference between this area and its surrounding areas, wrote as 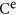 (estimated average-concentration-difference-halved concentration), and 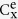 is the 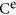 of a certain pollutant (x). For convenience, 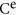 can be abbreviated to C.


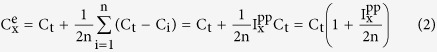


The explanations of x, i, n are same with those in the definition of PPI. In short, the 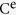 is the original concentration plus the average concentration difference between target and circum areas divided by two. Here, we split the average concentration difference fifty-fifty, treating the target area and its circum areas as two parallel lines.

The mathematical definition of Concentration Difference (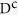) is represented in Equation 3. 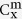 means the measured concentration of a pollutant x. 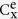 is defined in formula (2). 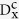 is the 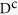 of a certain pollutant (x), which means the difference of the pollutant’s measured concentration minus its estimated average-concentration-difference-halved concentration. For convenience, 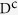 can be called D for short.


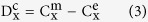


Equation 4 represents the mathematical definition of Overestimation or Underestimation Percentage (P^O/U^) of self-made pollution severity. 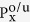 is the P^O/U^ of a certain pollutant (x), which is the quotient of the pollutant’s concentration difference to its estimated average-concentration-difference-halved concentration multiplied by 100%. For convenience, P^O/U^ can be abbreviated to P.


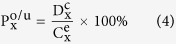


## Results and Discussion

**Figure 2:**
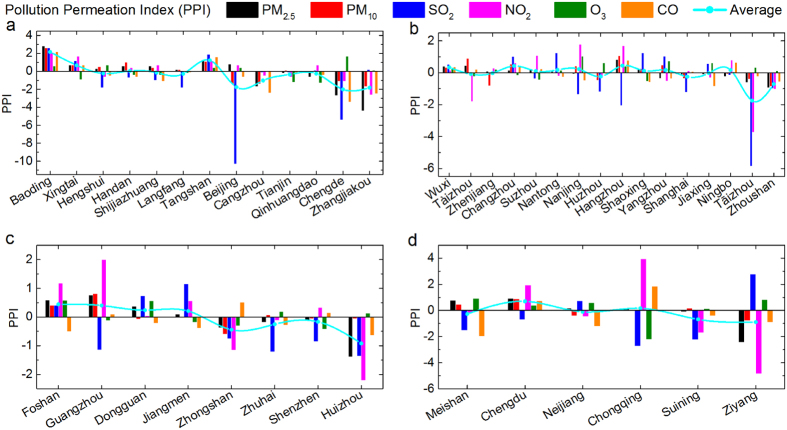
Pollution Permeation Index (PPI) of China’s four regions in 2015. (**a**) Jing-Jin-Ji (Beijing-Tianjin-Hebei), (**b**) Yangtze River Delta (YRD), (**c**) Pearl River Delta (PRD), and (**d**) Cheng-Yu (Chengdu-Chongqing). The specific PPI values are presented in Table S3.

The city sequence from left to right in Fig. 2 is listed in decreasing order of the PM_2.5_ concentrations in each region. Figure 2 shows cities with higher PM_2.5_ concentrations generally have positive (>0) PM_2.5_ PPI, but not in linear ways. Most cities have both positive and negative PPIs for different pollutants. When cities are judged on the basis of average PPI index (values in brackets), Baoding (2.15), Tangshan (1.22), Xingtai (0.68), and Handan (0.05) are the air pollution donors (underestimated or loosened) on the whole (regarding the six monitored regular pollutants) in Jing-Jin-Ji. It is found that Chengde (−2.01), Zhangjiakou (−1.82), Beijing (−1.76), Cangzhou (−1.02), Tianjin (−0.34), Langfang (−0.27), Qinhuangdao (−0.26), Hengshui (−0.23), and Shijiazhuang (−0.15) are air pollution receptors (overestimated or wronged) on the whole.

Figure 2 shows that SO_2_ has the greatest sum of absolute PPI values (in brackets) in Jing-Jin-Ji: SO_2_ (27.3) > PM_10_ (16.7) > CO (16.2) > NO_2_ (12.9) > PM_2.5_ (12.4) > O_3_ (8.2). The sum of absolute PPI value indicates the regional transport intensity (or degree) of a pollutant. Hence, in this region SO_2_, PM_10_, and CO are more susceptible to regional transport than NO_2_, PM_2.5_, or O_3_. For example, the SO_2_ PPI in Beijing (−10.35) is very low, which shows that SO_2_ is transported to Beijing during the study period.

Many more instructive results can be obtained from the data in Table S3 and their derivative data (such as absolute values). PPI users can analyze the air pollution status in details by using these data or by results from other novel PPI based methods that users develop. As the present study aims at introducing in general the analytical methods based on the PPI that is proposed, only limited results are presented for demonstration.

Researchers can use the customized ways in Table 1 to show the air pollution status of a target city and surrounding cities to the government and the public, when simple but efficient presentations are needed. Table 1 vividly inform readers for which pollutants a city can be called a pollution donor and or a pollution receptor in its region. As a whole, Beijing and Shanghai are pollution receptors while Guangzhou and Chengdu are pollution donors. The common point is that these four cities are pollution receptors in SO_2_ and pollution donors in NO_2_. The SO_2_ PPIs of Beijing, Shanghai, Guangzhou, and Chengdu are −10.35, −1.22, −1.14, and −0.70 respectively. Their NO_2_ PPIs are 0.68, 0.11, 2.00, and 1.95 respectively. This common feature demonstrates that these large populous cities tend to be infected by introduced SO_2_ from outside (regional sources such as coal) and have invasiveness of NO_2_ due to huge numbers of local vehicles.

**Figure 3:**
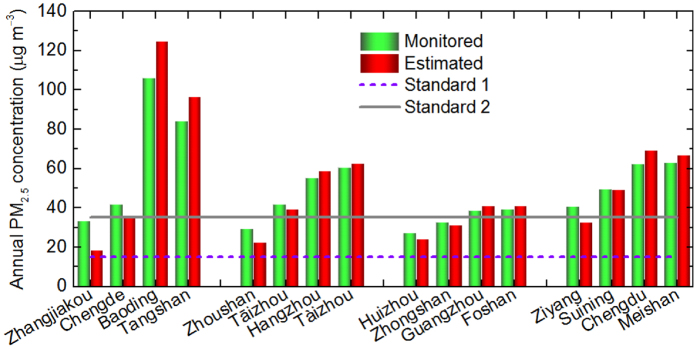
Examples of estimated annual PM_2.5_ concentrations in 2015 using PPI. Two biggest PM_2.5_ pollution donors and two biggest PM_2.5_ receptors in each region are shown here. Estimated concentrations of all the 6 regular pollutants PM_2.5_, PM_10_, SO_2_, NO_2_, O_3_, and CO by PPI in the exampled cities in 2015 are shown in Table S4.

Figure 3 shows the estimated average-concentration-difference-halved concentration (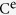) of PM_2.5_ in 16 exampled cities. Given fifty-fifty withdrawn circum influence (see Equation 2 for calculation method), some cities like Chengde and Ziyang should have met the level-2 of Chinese annual averaged PM_2.5_ standard; moreover, the PM_2.5_ concentrations in Zhangjiakou and Zhoushan should have been close to the annual averaged PM_2.5_ standard 1 of China.

Since the PPI calculated above is only based on the final results of annual averaged monitoring data, other variables such as geography and meteorology are ignored. Therefore, there is a scope of future work to improve the accuracy considering those variables.

**Figure 4:**
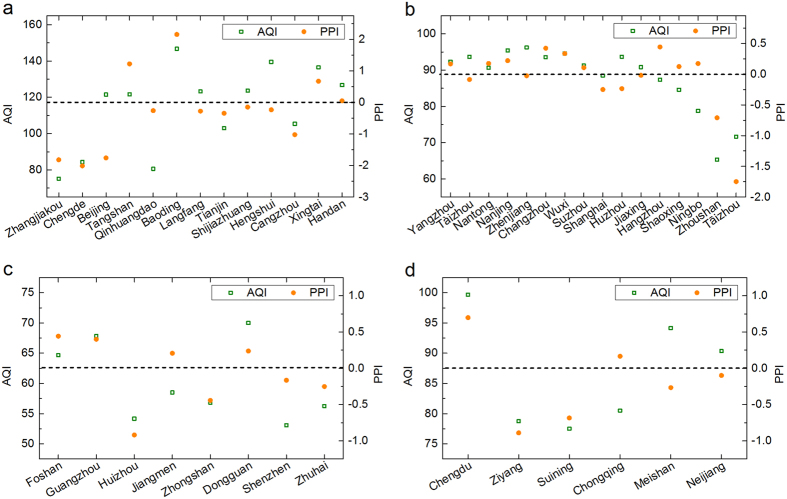
Comparison between AQI and PPI. (**a**) Jing-Jin-Ji (Beijing-Tianjin-Hebei), (**b**) Yangtze River Delta (YRD), (**c**) Pearl River Delta (PRD), and (**d**) Cheng-Yu (Chengdu-Chongqing). These two different indicators have similar tendencies, but the advantage of PPI is that it is capable of distinguishing an air pollution donor from an air pollution receptor. Moreover, a higher AQI does not necessarily imply an air pollution donor and vice versa.

Both Air Quality Index (AQI) and PPI are indexes. The comparison of those indexes is carried out, where the intriguing result is found. AQI can tell whether a city is heavily polluted or not, but it cannot tell whether a city is overall giving pollution or receiving pollution as PPI can do (Fig. 4). For example, Beijing, Langfang, Shijiazhuang, and Hengshui have relatively high annual average AQIs above 120 in Jing-Jin-Ji during 2015, but their PPIs (below 0) show they are more or less wrongly treated due to their features of pollution receptor. The PPI (below 0, as a role of receptor) in Hengshui might depend heavily on the relatively high level of pollution in Baoding (with higher concentrations of PM_2.5_, PM_10_, SO_2_, NO_2_ and CO) and Xingtai (with higher concentrations of PM_2.5_, SO_2_, NO_2_ and CO) as the surrounding area of Hengshui (Table S1). It is also found that Tàizhou, Shanghai and Huzhou in YRD, and Meishan and Neijiang in Cheng-Yu are also wronged. On the other hand, Ningbo in YRD, Jiangmen in PRD, and Chongqing in Cheng-Yu have relatively low AQIs, but they are loosened because of their roles of pollution donors judged by PPIs above 0.

**Figure 5:**
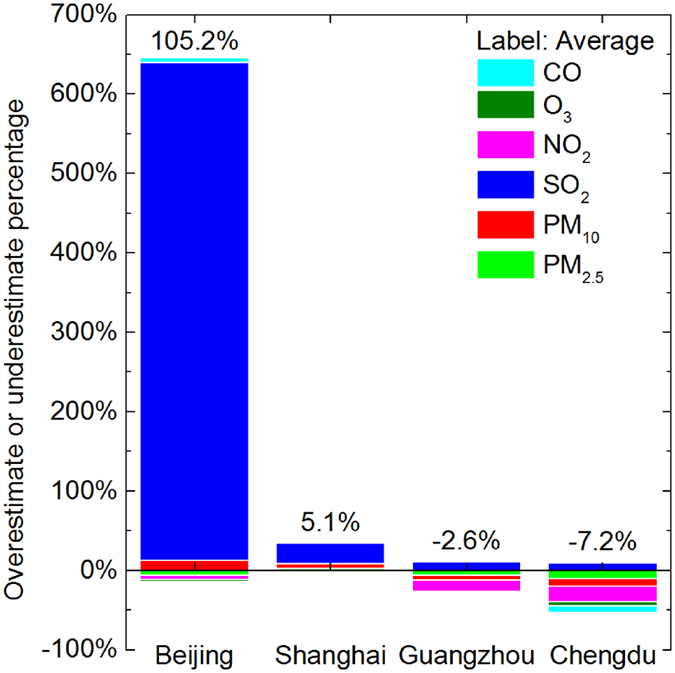
Overestimation or Underestimation Percentage (P^O/U^) of self-made pollution severity in 4 exampled cities in 2015. The reported percentage (label) is the average of the six pollutants’ P^O/U^ values. The concentration difference can be found in Table S5, and the overestimation or underestimation percentage in Table S6.

The results and discussions made above shows that PPI can tell whether the severity of self-made pollution is overestimated or underestimated. Furthermore, the overestimations and underestimations can be quantified by P^O/U^ (Fig. 5), a relative magnitude of the concentration difference between measured and estimated concentrations (Table S5). For example, the transportation of SO_2_ from neighboring cities to Beijing is very severe, because SO_2_ is 627.2% overestimated (Fig. 5). Hence, besides the local abundant NO_*x*_ (NO_2_ is 5.4% underestimated) and other related pollutants, the relatively high concentration of PM_2.5_ (6% underestimated) can be easily originated in Beijing (Table S6). In other words, higher PM_2.5_ mass concentration in Beijing is more indirectly influenced by the transported SO_2_ than by the direct transport of PM_2.5_ from neighboring cities. In this way, the PPI based method helps reveal the formation mechanism or process of PM_2.5_ pollution.

The validation study could be carried out in future with the results from other studies. However, it is not fully rational because there lies a controversy among different results as well. For example, some studies support our results^63^, which concluded that chemistry of urban traffic related VOCs and NO_*x*_ and regional SO_2_ contributed largely to PM_2.5_ in Beijing during September‒November 2013, but the influence of primary emissions and regional transported PM_2.5_ is small. However, this is surprisingly inconsistent with a broadly held view that the regional transported PM_2.5_ is a major source of smog in Beijing^64^. In other city like Shanghai, it is reported that PM_2.5_ were significantly due to local sources in the YRD^65^, instead of long-time transport from the Circum-Bohai-Sea (CBS) and northwestern China during 2011‒2012. Such study supports our work that focuses on surrounding influence and shows Shanghai was permeated PM_2.5_ (with a PPI of −0.12) pollution by its surrounding cities.

It is an omnipresent conclusion or a consensus that PM_2.5_ concentrations in big cities are a result of transport of pollutants from regional sources. However, unlike the present work, many other studies did not investigate the net influence (net result of interaction instead of one-way influence) a city gets from its all surrounding cities, which is unfavorable for the comparisons between current study and other studies. Most of the studies focus on the influence a target city can have from neighboring cities and other sources than otherwise. Hence, consideration of net effect of interaction or two-way influence in a region provides profound understanding on annual regional air quality. In such a way, other studies and current study would provide mutual validations.

Thus, it is highlighted that PPI is unable to accurately quantify the whole transport of air pollution, but it indicates the relative transport magnitude of different pollutants for each city. Accordingly, the PM_2.5_ PPI (positive except Shanghai) does not emphasize the direct regional sources of PM_2.5_ in the four big cities (Fig. 5, Tables 1 and S3), but the indirect regional sources of PM_2.5_ is supported by the introduction of SO_2_ from surrounding cities (negative PPIs) that jointly improves PM_2.5_ concentrations with self-emitted NO_2_ (positive PPIs).

## Limitations of the Method

The current study is only based on the annual averaged value of monitoring data. Thus, the method is limited for annual averaged monitoring results of pollutants only. When it comes to high temporal resolution, it can still show the potential of pollution permeation donor and receptor especially during heavy haze episodes that often concurrent with stable weather, but it still will have difficulty in reflecting timely process of pollution formation.

Concentration difference (infiltration), pressure (wind and turbulence), chemical reaction, physical boundary layer, and many other factors affect the diffusion and transport of air pollution. The independent variable (concentration) of PPI depends upon above factors. Thus, the actual concentration-dependent PPI itself is influenced by all the factors. Therefore, some other limitations of this method are: (1) the PPI is nonlinear, but the PPI based backward deduction in current study was linearly processed; (2) it only discusses about the penetration effect caused by concentration difference, thus it is unable to fully discuss the diffusion and transport of air pollution. However, all the final results are decided by the process of pollution formation, so the results-based PPI is essentially process-decided. Therefore, despite these limitations, the PPI can be considered as one of the valid and robust indexes in assessing regional air quality.

## Conclusions

The study concludes that PPI method can be used to distinguish the pollution permeation donor from the pollution permeation receptor by using the corresponding comparison of pollutant concentration. As shown in examples in the study, the PPI based circum comparison can be a convenient and quick method to assess the air pollution in different regions.

The study concludes the basic functions of this method as follows:

1. PPI itself can theoretically judge the pollution permeation potential (invasion ability). Positive PPI values correspond to air pollution permeation donors and negative PPIs to air pollution permeation receptors.
2. When pollution permeation (concentration-difference-driven circum influence) is halved as one part equal to the target area and another to its circum areas, the estimated average-concentration-difference-halved concentration (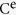) can be obtained. If the pollution permeation is withdrawn fifty-fifty, 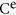 acts as the concentration that an area would have, reflecting the area’s inherent or self-made pollution status.
3. The method also helps to understand the formation mechanism or process that could affect different air pollutions (such as for PM_2.5_ pollution by SO_2_ and NO_2_). The overestimation or underestimation percentage (P^O/U^) of self-made pollution severity helps to apportion the local and non-local sources. With this quantification, the role and interactions between the internal and external sources can be partly assessed, which helps to identify the formation, causes and processes of pollution.

In addition, the PPI based tool is expected to find more applications in future. For example, health impact of air pollution may also be included in the applications of PPI based method. Furthermore, quantification and monetizing the health burden that an area contributing to or receiving from its surrounding areas could be assessed.

Hence, this method assesses regional air quality only based on pollutant concentration comparison, just like assessing the quality of a group of things by comparing performance instead of producing process. Therefore, it can identify the donors and receptors of pollution permeation rather than the process (formation, diffusion and transport) of pollution. An interesting phenomena of this method is that a combination of the PPIs of different pollutants can help to explain the formation, diffusion and transport of pollutants of a region in a quicker way.

## Additional Information

**How to cite this article**: Liang, C.-S. *et al*. Assessment of regional air quality by a concentration-dependent Pollution Permeation Index. *Sci. Rep*. **6**, 34891; doi: 10.1038/srep34891 (2016).

## Supplementary Material

Supplementary Information

## Figures and Tables

**Table 1:**
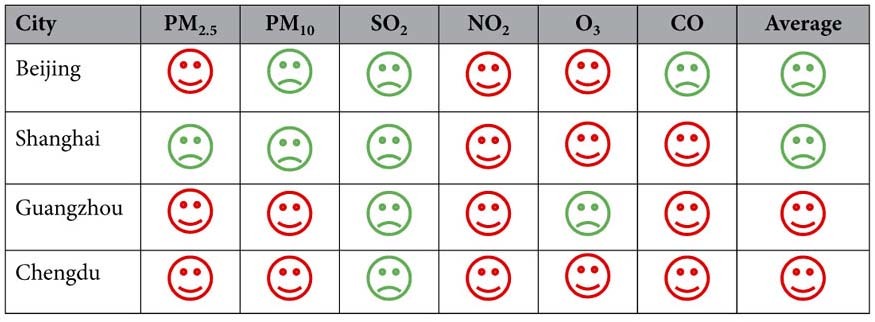
Examples of air pollution permeation donors and receptors in 2015 judged by Pollution Permeation Index.

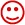
, donor; 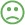, receptor.

## References

[b1] Pope R. & Wu J. G. A multi-objective assessment of an air quality monitoring network using environmental, economic, and social indicators and GIS-based. J Air Waste Manage 64, 721–737, doi: 10.1080/10962247.2014.888378 (2014). 25039205

[b2] Liang C.-S., Duan F.-K., He K.-B. & Ma Y.-L. Review on recent progress in observations, source identifications and countermeasures of PM_2.5_. Environ Int 86, 150–170, doi: 10.1016/j.envint.2015.10.016 (2016). 26595670

[b3] Demerjian K. L. A review of national monitoring networks in North America. Atmos Environ 34, 1861–1884, doi: 10.1016/s1352-2310(99)00452-5 (2000).

[b4] Stein A. F. . NOAA’s HYSPLIT Atmospheric Transport and Dispersion Modeling System. Bulletin of the American Meteorological Society 96, 2059–2077, 10.1175/BAMS-D-14-00110.1 (2015).

[b5] Pawar H. . Quantifying the contribution of long-range transport to particulate matter (PM) mass loadings at a suburban site in the north-western Indo-Gangetic Plain (NW-IGP). Atmos Chem Phys 15, 9501–9520, doi: 10.5194/acp-15-9501-2015 (2015).

[b6] Li T. . Concentrations and solubility of trace elements in fine particles at a mountain site, southern China: regional sources and cloud processing. Atmos Chem Phys 15, 8987–9002, doi: 10.5194/acp-15-8987-2015 (2015).

[b7] Pongkiatkul P. & Oanh N. T. K. Assessment of potential long-range transport of particulate air pollution using trajectory modeling and monitoring data. Atmos Res 85, 3–17, doi: 10.1016/j.atmosres.2006.10.003 (2007).

[b8] Byun D. & Schere K. L. Review of the governing equations, computational algorithms, and other components of the models-3 Community Multiscale Air Quality (CMAQ) modeling system. Appl. Mech. Rev. 59, 51–77, doi: 10.1115/1.2128636 (2006).

[b9] Eder B. & Yu S. C. A performance evaluation of the 2004 release of Models-3 CMAQ. Atmos Environ 40, 4811–4824, doi: 10.1016/j.atmosenv.2005.08.045 (2006).

[b10] Mueller S. F. & Mallard J. W. Contributions of Natural Emissions to Ozone and PM_2.5_ as Simulated by the Community Multiscale Air Quality (CMAQ) Model. Environ Sci Technol 45, 4817–4823, doi: 10.1021/es103645m (2011). 21545154

[b11] Wang L. T. . The 2013 severe haze over southern Hebei, China: model evaluation, source apportionment, and policy implications. Atmos Chem Phys 14, 3151–3173, doi: 10.5194/acp-14-3151-2014 (2014).

[b12] Tian D. . Assessment of Biomass Burning Emissions and Their Impacts on Urban and Regional PM_2.5_: A Georgia Case Study. Environ Sci Technol 43, 299–305, doi: 10.1021/es801827s (2009). 19238955

[b13] Chemel C. . Predictions of UK Regulated Power Station Contributions to Regional Air Pollution and Deposition: A Model Comparison Exercise. J Air Waste Manage 61, 1236–1245, doi: 10.1080/10473289.2011.609756 (2011). 22168107

[b14] Sun Y. L. . Long-term real-time measurements of aerosol particle composition in Beijing, China: seasonal variations, meteorological effects, and source analysis. Atmos Chem Phys 15, 10149–10165, doi: 10.5194/acp-15-10149-2015 (2015).

[b15] Lee P. K. H., Brook J. R., Dabek-Zlotorzynska E. & Mabury S. A. Identification of the major sources contributing to PM_2.5_ observed in Toronto. Environ Sci Technol 37, 4831–4840, doi: 10.1021/es026473i (2003). 14620807

[b16] Kim E., Hopke P. K., Pinto J. P. & Wilson W. E. Spatial variability of fine particle mass, components, and source contributions during the regional air pollution study in St. Louis. Environ Sci Technol 39, 4172–4179, doi: 10.1021/es049824x (2005). 15984797

[b17] Hwang I., Hopke P. K. & Pinto J. P. Source apportionment and spatial distributions of coarse particles during the regional air pollution study. Environ Sci Technol 42, 3524–3530, doi: 10.1021/es0716204 (2008). 18546684

[b18] Tang L. L. . Regional contribution to PM_1_ pollution during winter haze in Yangtze River Delta, China. Sci Total Environ 541, 161–166, doi: 10.1016/j.scitotenv.2015.05.058 (2016). 26414850

[b19] Xie M. J. . Positive Matrix Factorization of PM_2.5_: Comparison and Implications of Using Different Speciation Data Sets. Environ Sci Technol 46, 11962–11970, doi: 10.1021/es302358g (2012). 22985292

[b20] Kotchenruther R. A. A regional assessment of marine vessel PM_2.5_ impacts in the U.S. Pacific Northwest using a receptor-based source apportionment method. Atmos Environ 68, 103–111, doi: 10.1016/j.atmosenv.2012.11.067 (2013).

[b21] Sfetsos A. & Vlachogiannis D. An analysis of ozone variation in the Greater Athens Area using Granger Causality. Atmos Pollut Res 4, 290–297, doi: 10.5094/apr.2013.032 (2013).

[b22] de Paula P. H. M. . Biomonitoring of metals for air pollution assessment using a hemiepiphyte herb (Struthanthus flexicaulis). Chemosphere 138, 429–437, doi: 10.1016/j.chemosphere.2015.06.060 (2015). 26160299

[b23] Xue R. . Spatial distribution and source apportionment of PAHs in marine surface sediments of Prydz Bay, East Antarctica. Environ Pollut, doi: 10.1016/j.envpol.2016.05.084 (2016). 27318541

[b24] Zhang Q. Q. . Regional differences in Chinese SO2 emission control efficiency and policy implications. Atmos Chem Phys 15, 6521–6533, doi: 10.5194/acp-15-6521-2015 (2015).

[b25] Zhang Q. J. . Formation of secondary organic aerosol in the Paris pollution plume and its impact on surrounding regions. Atmos Chem Phys 15, 13973–13992, doi: 10.5194/acp-15-13973-2015 (2015).

[b26] Zhang L. . Sources contributing to background surface ozone in the US Intermountain West. Atmos Chem Phys 14, 5295–5309, doi: 10.5194/acp-14-5295-2014 (2014).

[b27] Thomas J. L. . Pollution transport from North America to Greenland during summer 2008. Atmos Chem Phys 13, 3825–3848, doi: 10.5194/acp-13-3825-2013 (2013).

[b28] Skyllakou K., Murphy B. N., Megaritis A. G., Fountoukis C. & Pandis S. N. Contributions of local and regional sources to fine PM in the megacity of Paris. Atmos Chem Phys 14, 2343–2352, doi: 10.5194/acp-14-2343-2014 (2014).

[b29] Kulkarni S. . Source sector and region contributions to BC and PM_2.5_ in Central Asia. Atmos Chem Phys 15, 1683–1705, doi: 10.5194/acp-15-1683-2015 (2015).

[b30] Huszar P., Belda M. & Halenka T. On the long-term impact of emissions from central European cities on regional air quality. Atmos Chem Phys 16, 1331–1352, doi: 10.5194/acp-16-1331-2016 (2016).

[b31] Bian H. . Source attributions of pollution to the Western Arctic during the NASA ARCTAS field campaign. Atmos Chem Phys 13, 4707–4721, doi: 10.5194/acp-13-4707-2013 (2013).

[b32] Beekmann M. . *In situ*, satellite measurement and model evidence on the dominant regional contribution to fine particulate matter levels in the Paris megacity. Atmos Chem Phys 15, 9577–9591, doi: 10.5194/acp-15-9577-2015 (2015).

[b33] Monteiro A., Miranda A. I., Borrego C. & Vautard R. Air quality assessment for Portugal. Sci Total Environ 373, 22–31, doi: 10.1016/j.scitotenv.2006.10.014 (2007). 17207847

[b34] Kiesewetter G. . Modelling NO2 concentrations at the street level in the GAINS integrated assessment model: projections under current legislation. Atmos Chem Phys 14, 813–829, doi: 10.5194/acp-14-813-2014 (2014).

[b35] Tao W. . Effects of urban land expansion on the regional meteorology and air quality of eastern China. Atmos Chem Phys 15, 8597–8614, doi: 10.5194/acp-15-8597-2015 (2015).

[b36] Liu D. . The importance of Asia as a source of black carbon to the European Arctic during springtime 2013. Atmos Chem Phys 15, 11537–11555, doi: 10.5194/acp-15-11537-2015 (2015).

[b37] Souto J. A. . PRESAXIO regional air quality modelling system: validation and applications. Int J Environ Pollut 55, 192–200, doi: 10.1504/ijep.2014.065924 (2014).

[b38] Marcon A., de Hoogh K., Gulliver J., Beelen R. & Hansell A. L. Development and transferability of a nitrogen dioxide land use regression model within the Veneto region of Italy. Atmos Environ 122, 696–704, doi: 10.1016/j.atmosenv.2015.10.010 (2015).

[b39] Chen L. . A land use regression model incorporating data on industrial point source pollution. J. Environ. Sci. 24, 1251–1258, doi: 10.1016/s1001-0742(11)60902-9 (2012). 23513446

[b40] Hoek G. . A review of land-use regression models to assess spatial variation of outdoor air pollution. Atmos Environ 42, 7561–7578, doi: 10.1016/j.atmosenv.2008.05.057 (2008).

[b41] Schulte J. K. . Neighborhood-Scale Spatial Models of Diesel Exhaust Concentration Profile Using 1-Nitropyrene and Other Nitroarenes. Environ Sci Technol 49, 13422–13430, doi: 10.1021/acs.est.5b03639 (2015). 26501773 PMC5026850

[b42] Mouzourides P., Kumar P. & Neophytou M. K. A. Assessment of long-term measurements of particulate matter and gaseous pollutants in South-East Mediterranean. Atmos Environ 107, 148–165, doi: 10.1016/j.atmosenv.2015.02.031 (2015).

[b43] Xue L. K. . Increasing External Effects Negate Local Efforts to Control Ozone Air Pollution: A Case Study of Hong Kong and Implications for Other Chinese Cities. Environ Sci Technol 48, 10769–10775, doi: 10.1021/es503278g (2014). 25133661

[b44] Wang B. Z. & Chen Z. A GIS-based multi-source and multi-box modeling approach (GMSMB) for air pollution assessment-A North American case study. J Environ Sci Heal A 48, 14–25, doi: 10.1080/10934529.2012.707597 (2013). 23030384

[b45] Chen S. . Global Sensitivity Analysis of the Regional Atmospheric Chemical Mechanism: An Application of Random Sampling-High Dimensional Model Representation to Urban Oxidation Chemistry. Environ Sci Technol 46, 11162–11170, doi: 10.1021/es301565w (2012). 22963531

[b46] Zheng G. . Episode-Based Evolution Pattern Analysis of Haze Pollution: Method Development and Results from Beijing, China. Environ Sci Technol 50, 4632–4641, doi: 10.1021/acs.est.5b05593 (2016). 27050081

[b47] Mayer H. & Kalberlah F. Two impact related air quality indices as tools to assess the daily and long-term air pollution. Int J Environ Pollut 36, 19–29, doi: 10.1504/ijep.2009.021814 (2009).

[b48] Cheng W. L. . Comparison of the Revised Air Quality Index with the PSI and AQI indices. Sci Total Environ 382, 191–198, doi: 10.1016/j.scitotenv.2007.04.036 (2007). 17540435

[b49] Cairncross E. K., John J. & Zunckel M. A novel air pollution index based on the relative risk of daily mortality associated with short-term exposure to common air pollutants. Atmos Environ 41, 8442–8454, doi: 10.1016/j.atmosenv.2007.07.003 (2007).

[b50] Trivero P. . An air quality balance index estimating the total amount of air pollutants at ground level. Environ Monit Assess 184, 4461–4472, doi: 10.1007/s10661-011-2278-1 (2012). 21830066

[b51] Zheng J. Y., Feng X. Q., Liu P. W., Zhong L. J. & Lai S. C. Site location optimization of regional air quality monitoring network in china: methodology and case study. J. Environ. Monit. 13, 3185–3195, doi: 10.1039/c1em10560d (2011). 22006403

[b52] Zhao L. J., Xie Y. J., Wang J. J. & Xu X. A performance assessment and adjustment program for air quality monitoring networks in Shanghai. Atmos Environ 122, 382–392, doi: 10.1016/j.atmosenv.2015.09.069 (2015).

[b53] Kulmala M. Atmospheric chemistry: China’s choking cocktail. Nature 526, 497–499 (2015). 26490602 10.1038/526497a

[b54] Wang X. K. & Lu W. Z. Seasonal variation of air pollution index: Hong Kong case study. Chemosphere 63, 1261–1272, doi: 10.1016/j.chemosphere.2005.10.031 (2006). 16325232

[b55] Ozden O., Dogeroglu T. & Kara S. Assessment of ambient air quality in Eskisehir, Turkey. Environ Int 34, 678–687, doi: 10.1016/j.envint.2007.12.016 (2008). 18291527

[b56] Miranda A. . Current air quality plans in Europe designed to support air quality management policies. Atmos Pollut Res 6, 434–443, doi: 10.5094/apr.2015.048 (2015).

[b57] Janssen S. . Land use to characterize spatial representativeness of air quality monitoring stations and its relevance for model validation. Atmos Environ 59, 492–500, doi: 10.1016/j.atmosenv.2012.05.028 (2012).

[b58] Toro A. R., Campos C., Molina C., Morales R. G. E. & Leiva-Guzman M. A. Accuracy and reliability of Chile’s National Air Quality Information System for measuring particulate matter: Beta attenuation monitoring issue. Environ Int 82, 101–109, doi: 10.1016/j.envint.2015.02.009 (2015). 25796098

[b59] Jiao W. . Field Assessment of the Village Green Project: An Autonomous Community Air Quality Monitoring System. Environ Sci Technol 49, 6085–6092, doi: 10.1021/acs.est.5b01245 (2015). 25905923

[b60] Kumar P. . The rise of low-cost sensing for managing air pollution in cities. Environ Int 75, 199–205, doi: 10.1016/j.envint.2014.11.019 (2015). 25483836

[b61] MEPPRC. National Urban Ambient Air Quality Daily Report (Ministry of Environmental Protection of the People’s Republic of China). http://datacenter.mep.gov.cn/; http://www.gov.cn/xinwen/2015-01/16/content_2805618.htm January (2015).

[b62] USEPA. PM_2.5_ Objectives and History. https://archive.epa.gov/pesticides/region4/sesd/pm25/web/html/p2.html February (2016).

[b63] Guo S. . Elucidating severe urban haze formation in China. Proceedings of the National Academy of Sciences 111, 17373–17378, doi: 10.1073/pnas.1419604111 (2014). PMC4267398 25422462

[b64] Li P. . Reinstate regional transport of PM_2.5_ as a major cause of severe haze in Beijing. Proceedings of the National Academy of Sciences 112, E2739–E2740, doi: 10.1073/pnas.1502596112 (2015). PMC4450418 25941410

[b65] Zhao M. F. . Chemical characterization, the transport pathways and potential sources of PM_2.5_ in Shanghai: Seasonal variations. Atmos Res 158, 66–78, doi: 10.1016/j.atmosres.2015.02.003 (2015).

