# Supplementary material for: Assessment of regional air quality by a concentration-dependent Pollution Permeation Index
Source: Sci Rep. 2016 Oct 12;6:34891. doi: 10.1038/srep34891 (PMC5059628; doi:10.1038/srep34891)
Supplement: Supplementary Information [file srep34891-s1.pdf]

Supplementary Information:

Assessment of regional air quality by a concentration-dependent Pollution Permeation Index

Chun-Sheng Liang[1,3], Huan Liu[1,2,*], Ke-Bin He[1,2,*] & Yong-Liang Ma[1,2]

[1] State Key Joint Laboratory of Environment Simulation and Pollution Control, School of Environment, Tsinghua University, Beijing 100084, China

[2] State Environmental Protection Key Laboratory of Sources and Control of Air Pollution Complex, Tsinghua University, Beijing 100084, China

[3] Institute of Atmospheric Environment, Chinese Research Academy of Environmental Sciences, Beijing 100012, China

This file contains Tables S1–S6.

Table S1. Yearly air pollution data of the exampled cities and their neighbor cities in 2015

City	AQI	$PM_{2.5}$	PM_{10}	SO_2	NO_2	O_3	CO	Ranking
**Jing-Jin-Ji**								
Zhangjiakou	75	33.3	79.5	32.4	25.5	99.2	0.84	170
Chengde	84	41.8	86.9	21.4	33.1	101.6	1.01	188
Beijing	122	80.2	100.5	12.8	49.0	99.2	1.30	245
Tangshan	122	84.2	140.6	48.6	60.2	94.5	2.13	278
Qinhuangdao	81	47.0	97.8	37.9	45.3	65.0	1.48	184
Baoding	147	106.2	175.3	54.7	53.6	99.2	1.88	296
Langfang	123	84.7	137.7	23.6	46.7	87.5	1.41	264
Tianjin	103	69.8	120.3	28.9	41.4	76.8	1.37	237
Shijiazhuang	124	88.1	147.7	48.6	49.4	79.1	1.37	270
Hengshui	140	98.6	175.0	36.2	43.6	107.9	1.50	308
Cangzhou	105	69.9	121.4	40.0	41.2	93.9	1.22	249
Xingtai	137	99.8	171.3	60.5	59.4	78.6	1.80	298
Handan	127	90.7	167.2	45.4	46.7	80.0	1.60	295
**Circum cities outside Jing-Jin-Ji**								
Wulanchabu	81	45.2	77.8	25.1	30.8	102.2	0.73	188
Chifeng	74	41.3	85.6	48.7	24.7	61.6	1.02	160
Chaoyang	70	39.7	73.8	28.0	19.0	58.4	1.85	152
Huludao	91	53.1	97.0	48.7	36.5	90.1	1.51	219
Binzhou	111	79.0	124.9	57.2	42.1	82.1	2.10	256

Dezhou	141	100.3	164.1	43.5	42.2	110.8	1.96	308
Liaocheng	134	98.3	159.9	42.1	42.2	95.8	1.72	305
Puyang	116	81.3	140.3	29.9	41.3	91.6	1.60	275
Anyang	125	90.7	150.0	50.3	48.8	75.1	2.11	284
Changzhi	97	64.3	106.0	49.3	37.3	104.1	1.96	237
Jinzhong	88	56.8	100.4	72.1	31.6	67.7	1.42	212
Yangquan	90	53.9	113.4	59.2	40.2	73.3	1.31	213
Xinzhou	88	58.4	91.0	57.1	35.4	83.3	1.87	194
Datong	79	39.9	88.3	42.0	26.6	87.5	1.37	188
**YRD**								
Yangzhou	92	54.3	100.4	24.8	30.0	108.5	0.89	230
Tàizhou	94	60.6	103.4	24.7	30.5	100.7	0.96	230
Nantong	91	57.0	86.2	29.2	36.1	108.0	0.82	214
Nanjing	95	56.6	96.6	19.1	49.8	101.3	0.97	233
Zhenjiang	96	59.1	83.7	25.0	41.5	106.6	0.98	233
Changzhou	94	58.9	102.0	29.7	44.3	93.7	1.08	229
Wuxi	95	61.2	94.0	25.9	41.2	99.9	1.05	231
Suzhou	91	57.8	80.9	20.4	52.5	97.8	0.92	222
Shanghai	89	53.6	72.9	16.6	45.6	105.4	0.86	207
Huzhou	94	55.5	75.2	17.2	40.4	108.7	0.91	218
Jiaxing	91	52.7	76.7	20.6	43.2	109.7	0.76	214
Hangzhou	87	55.4	83.2	15.5	46.3	94.3	0.90	210
Shaoxing	85	54.5	80.5	27.0	44.9	87.1	0.76	201
Ningbo	79	44.7	69.5	15.5	42.7	99.3	0.89	178
Zhoushan	65	29.4	47.1	8.0	21.7	103.7	0.66	129
Tāizhou	72	42.0	64.7	8.4	23.0	97.0	0.79	163
**Circum cities outside selected YRD cities**								
Chuzhou	85	62.1	89.1	14.5	28.7	42.9	0.96	205
Huaian	95	57.9	96.1	20.9	22.0	106.3	1.05	231
Yancheng	89	49.1	85.5	18.9	23.3	113.5	0.77	213
Maanshan	87	60.5	88.5	22.4	33.2	77.4	1.40	212
Xuancheng	75	48.4	76.3	24.0	32.2	74.7	1.00	169
Huangshan	51	34.7	47.3	8.9	13.7	53.2	0.22	84
Quzhou	71	42.7	66.4	20.1	31.9	96.2	1.03	162
Jinhua	84	54.4	72.0	22.7	39.9	93.0	0.93	201
Lishui	65	38.2	55.5	11.1	28.0	86.6	0.69	131
Wenzhou	72	43.7	71.5	14.8	44.7	87.1	0.90	171
**PRD**								
Foshan	65	39.3	58.9	17.0	40.2	81.6	0.93	135
Guangzhou	68	38.8	60.5	12.4	45.5	82.7	0.97	147
Huizhou	54	27.2	50.3	9.9	21.2	90.5	0.82	95
Jiangmen	59	34.5	53.0	16.0	31.5	73.1	0.92	112

Zhongshan	57	32.8	49.0	11.4	28.2	74.0	1.06	104
Dongguan	70	36.4	52.3	13.4	33.7	103.8	0.83	144
Shenzhen	53	29.7	49.8	8.2	33.0	80.3	0.89	94
Zhuhai	56	31.0	53.0	8.5	28.4	81.0	0.87	105
**Circum cities outside selected PRD cities**								
Qingyuan	57	33.6	52.7	17.3	33.9	74.7	1.06	102
Shaoguan	57	33.8	51.3	19.2	24.7	81.9	1.01	108
Heyuan	57	34.4	50.1	9.9	23.7	86.5	0.92	107
Shanwei	55	27.6	41.0	9.9	13.0	94.9	0.80	93
Zhaoqing	66	38.8	58.1	20.3	30.5	87.5	0.94	137
Yunfu	54	34.2	55.7	18.0	24.1	50.4	1.12	101
Yangjiang	58	32.2	48.5	6.7	18.6	91.0	0.99	106
**Cheng-Yu**								
Chengdu	100	62.4	105.1	15.3	50.7	94.6	1.10	238
Ziyang	79	40.7	80.2	30.2	19.0	101.4	0.80	182
Suining	78	49.5	85.7	12.8	23.3	83.9	0.88	181
Chongqing	81	55.0	85.2	16.0	43.7	65.7	1.11	187
Meishan	94	62.9	94.0	15.9	30.0	95.5	0.71	228
Neijiang	90	60.5	84.1	23.7	28.1	91.8	0.69	212
**Circum cities outside selected Cheng-Yu cities**								
Aba	30	14.2	29.7	3.6	5.7	55.0	0.20	5
Deyang	85	53.1	88.5	13.8	29.9	94.0	1.01	199
Mianyang	73	46.3	72.2	12.4	33.7	81.2	0.93	160
Nanchong	85	60.3	91.5	11.8	30.4	52.9	0.89	208
Guangan	84	46.0	81.9	20.9	21.9	97.1	0.91	188
Dazhou	88	62.9	90.3	11.0	38.8	59.1	1.08	209
Ankang	80	51.7	77.7	23.8	17.7	86.6	1.19	178
Shiyan	79	54.1	87.1	26.4	26.7	71.1	1.05	186
Zunyi	65	41.7	71.8	15.5	28.5	68.3	0.84	142
Luzhou	86	60.7	88.5	21.3	30.7	73.9	0.67	200
Zigong	101	73.6	107.7	17.9	30.1	68.0	0.96	240
Leshan	84	55.7	79.7	20.2	33.1	81.4	1.08	194
Yaan	58	36.2	65.4	12.0	25.0	49.1	1.05	110

- Data taken from http://www.aqistudy.cn/historydata/.

Table S2. Target and circum cities in Jing-Jin-Ji, and in the core areas of YRD, PRD and Cheng-Yu

**Target**	**Circum**	**Target**	**Circum**	**Target**	**Circum**	**Target**	**Circum**
**Jing-Jin-Ji**		**YRD**		**PRD**		**Cheng-Yu**	
Zhangjiakou	Wulanchabu	Yangzhou	Huaian	Foshan	Zhaoqing	Chengdu	Deyang
	Chengde		Yancheng		Qingyuan		Ziyang
	Beijing		Taizhou		Guangzhou		Meishan

	Baoding		Zhenjiang		Zhongshan		Yaan
	Datong		Nanjing		Jiangmen	Ziyang	Chengdu
Chengde	Zhangjiakou		Chuzhou		Yunfu		Deyang
	Chifeng	Tàizhou	Yangzhou	Guangzhou	Qingyuan		Suining
	Chaoyang		Yancheng		Shaoguan		Chongqing
	Huludao		Nantong		Huizhou		Neijiang
	Qinhuangdao		Suzhou		Dongguan		Meishan
	Tangshan		Wuxi		Zhongshan	Suining	Deyang
	Tianjin		Changzhou		Foshan		Mianyang
	Beijing		Zhenjiang	Huizhou	Guangzhou		Nanchong
Beijing	Zhangjiakou	Nantong	Tàizhou		Shaoguan		Guangan
	Chengde		Yancheng		Heyuan		Chongqing
	Tangshan		Shanghai		Shanwei		Ziyang
	Tianjin		Suzhou		Shenzhen	Chongqing	Ziyang
	Langfang	Nanjing	Chuzhou		Dongguan		Suining
	Baoding		Yangzhou	Jiangmen	Yunfu		Guangan
Tangshan	Beijing		Zhenjiang		Foshan		Dazhou
	Chengde		Changzhou		Zhongshan		Ankang
	Qinhuangdao		Xuancheng		Zhuhai		Shiyan
	Tianjin		Maanshan		Yangjiang		Zunyi
Qinhuangdao	Chengde	Zhenjiang	Nanjing	Zhongshan	Foshan		Luzhou
	Chaoyang		Yangzhou		Guangzhou		Zigong
	Huludao		Tàizhou		Zhuhai		Neijiang
	Tangshan		Changzhou		Jiangmen	Meishan	Yaan
Baoding	Datong	Changzhou	Nanjing	Dongguan	Guangzhou		Chengdu
	Zhangjiakou		Zhenjiang		Huizhou		Ziyang
	Beijing		Tàizhou		Shenzhen		Neijiang
	Langfang		Wuxi	Shenzhen	Dongguan		Zigong
	Cangzhou		Xuancheng		Huizhou		Leshan
	Hengshui	Wuxi	Changzhou	Zhuhai	Jiangmen	Neijiang	Meishan
	Shijiazhuang		Tàizhou		Zhongshan		Ziyang
	Xinzhou		Suzhou				Chongqing
Langfang	Beijing		Huzhou				Luzhou
	Tianjin		Xuancheng				Zigong
	Cangzhou	Suzhou	Wuxi				
	Baoding		Nantong				
Tianjin	Beijing		Shanghai				
	Chengde		Jiaxing				
	Tangshan		Huzhou				
	Cangzhou	Shanghai	Suzhou				
	Langfang		Nantong				
Shijiazhuang	Yangquan		Jiaxing				
	Xinzhou	Huzhou	Wuxi				

	Baoding		Suzhou				
	Hengshui		Jiaxing				
	Xingtai		Hangzhou				
	Jinzhong		Xuancheng				
Hengshui	Shijiazhuang	Jiaxing	Suzhou				
	Baoding		Shanghai				
	Cangzhou		Ningbo				
	Dezhou		Shaoxing				
	Xingtai		Hangzhou				
Cangzhou	Baoding		Huzhou				
	Langfang	Hangzhou	Xuancheng				
	Tianjin		Huzhou				
	Binzhou		Jiaxing				
	Dezhou		Shaoxing				
	Hengshui		Jinhua				
Xingtai	Jinzhong		Quzhou				
	Shijiazhuang		Huangshan				
	Hengshui	Shaoxing	Hangzhou				
	Dezhou		Jiaxing				
	Liaocheng		Ningbo				
	Handan		Jinhua				
Handan	Jinzhong	Ningbo	Hangzhou				
	Xingtai		Jiaxing				
	Liaocheng		Zhoushan				
	Puyang		Tāizhou				
	Anyang		Shaoxing				
	Changzhi	Zhoushan	Ningbo				
			Tāizhou				
		Tāizhou	Shaoxing				
			Ningbo				
			Wenzhou				
			Lishui				
			Jinhua				

Table S3. Calculated Pollution Permeation Index in 2015

City	$I_{PM_{2.5}}$	$I_{PM_{10}}$	I_{SO_2}	I_{NO_2}	I_{O_3}	I_{CO}	Average Index
**Jing-Jin-Ji**							
Zhangjiakou	-4.42	-1.65	0.18	-2.57	0.06	-2.50	-1.82
Chengde	-2.73	-1.15	-5.37	-1.11	1.66	-3.36	-2.01
Beijing	0.76	-1.36	-10.35	0.68	0.37	-0.64	-1.76
Tangshan	1.16	1.11	1.92	1.20	0.37	1.57	1.22
Qinhuangdao	-0.65	-0.07	0.13	0.71	-1.30	-0.37	-0.26

Baoding	2.79	2.63	2.65	2.08	0.56	2.21	2.15
Langfang	0.15	0.24	-1.77	0.03	-0.22	-0.08	-0.27
Tianjin	-0.17	0.12	-0.07	-0.55	-1.20	-0.18	-0.34
Shijiazhuang	0.62	0.40	-0.99	0.65	-0.44	-1.13	-0.15
Hengshui	0.29	0.54	-1.83	-0.64	0.72	-0.48	-0.23
Cangzhou	-1.71	-1.39	-0.10	-0.54	-0.01	-2.37	-1.02
Xingtai	0.66	0.66	1.24	1.70	-0.89	0.68	0.68
Handan	0.59	1.05	-0.69	0.42	-0.41	-0.65	0.05
**YRD**							
Yangzhou	-0.36	0.48	1.03	-0.52	0.73	-0.37	0.17
Tàizhou	0.45	0.88	-0.03	-1.81	-0.23	0.21	-0.09
Nantong	0.12	0.03	1.24	-0.21	0.14	-0.26	0.17
Nanjing	-0.07	0.41	-1.36	1.78	1.03	-0.48	0.22
Zhenjiang	0.10	-0.81	0.06	0.27	0.21	0.00	-0.03
Changzhou	0.14	0.55	1.00	0.60	-0.16	0.39	0.42
Wuxi	0.40	0.34	0.52	0.15	0.24	0.35	0.33
Suzhou	0.16	0.00	-0.38	1.07	-0.44	0.23	0.11
Shanghai	-0.12	-0.35	-1.22	0.11	0.01	0.07	-0.25
Huzhou	0.03	-0.47	-1.19	-0.34	0.62	-0.07	-0.24
Jiaxing	-0.10	-0.02	0.56	-0.30	0.60	-0.85	-0.02
Hangzhou	0.81	1.06	-2.06	1.68	0.40	0.77	0.44
Shaoxing	0.20	0.25	1.25	0.17	-0.55	-0.59	0.12
Ningbo	-0.24	-0.07	-0.14	0.81	0.05	0.62	0.17
Zhoushan	-0.94	-0.85	-0.99	-1.03	0.11	-0.56	-0.71
Tāizhou	-0.61	-0.40	-5.85	-3.72	0.33	-0.24	-1.75
**PRD**							
Foshan	0.58	0.41	0.41	1.18	0.58	-0.51	0.44
Guangzhou	0.77	0.81	-1.14	2.00	-0.12	0.10	0.40
Huizhou	-1.39	-0.06	-1.36	-2.21	0.14	-0.63	-0.92
Jiangmen	0.09	0.00	1.15	0.57	-0.17	-0.39	0.21
Zhongshan	-0.38	-0.60	-0.74	-1.15	-0.30	0.51	-0.44
Dongguan	0.37	-0.07	0.73	0.05	0.56	-0.21	0.24
Shenzhen	-0.14	-0.06	-0.85	0.34	-0.42	0.14	-0.16
Zhuhai	-0.17	0.08	-1.21	-0.11	0.18	-0.28	-0.25
**Cheng-Yu**							
Chengdu	0.91	0.88	-0.70	1.95	0.41	0.75	0.70
Ziyang	-2.43	-0.77	2.77	-4.85	0.82	-0.88	-0.89
Suining	-0.09	0.17	-2.23	-1.68	0.13	-0.41	-0.68
Chongqing	0.15	-0.03	-2.73	3.94	-2.20	1.86	0.17
Meishan	0.77	0.44	-1.52	-0.20	0.91	-1.99	-0.27
Neijiang	0.16	-0.42	0.73	-0.46	0.60	-1.21	-0.10

Table S4. Estimated concentrations by Pollution Permeation Index in 2015

City	$C_{PM_{2.5}}$	$C_{PM_{10}}$	C_{SO_2}	C_{NO_2}	C_{O_3}	C_{CO}
**Jing-Jin-Ji**						
Zhangjiakou	18.6	66.4	33.0	18.9	99.8	0.63
Chengde	34.7	80.7	14.2	30.8	112.2	0.80
Beijing	85.3	89.1	1.8	51.7	102.3	1.23
Tangshan	96.4	160.1	60.2	69.2	98.9	2.54
Qinhuangdao	43.2	96.9	38.5	49.3	54.4	1.41
Baoding	124.7	204.1	63.7	60.6	102.7	2.14
Langfang	86.3	141.8	18.4	46.8	85.1	1.40
Tianjin	68.6	121.7	28.7	39.1	67.6	1.34
Shijiazhuang	92.6	152.6	44.6	52.0	76.2	1.24
Hengshui	101.5	184.5	29.6	40.8	115.7	1.43
Cangzhou	60.0	107.3	39.6	39.3	93.8	0.98
Xingtai	105.3	180.8	66.7	67.8	72.7	1.90
Handan	95.2	181.8	42.8	48.3	77.3	1.51
**YRD**						
Yangzhou	52.7	104.3	26.9	28.7	115.1	0.87
Tàizhou	62.6	109.9	24.7	26.6	99.0	0.97
Nantong	57.8	86.5	33.7	35.1	109.8	0.80
Nanjing	56.3	100.0	16.9	57.2	110.0	0.93
Zhenjiang	59.8	75.2	25.2	42.9	109.4	0.98
Changzhou	59.7	107.5	32.7	47.0	92.2	1.12
Wuxi	63.6	97.2	27.3	41.8	102.3	1.08
Suzhou	58.7	80.9	19.6	58.1	93.5	0.94
Shanghai	52.5	68.6	13.2	46.5	105.5	0.87
Huzhou	55.6	71.7	15.1	39.0	115.4	0.91
Jiaxing	52.3	76.6	21.6	42.1	115.1	0.71
Hangzhou	58.6	89.5	13.2	51.8	97.0	0.95
Shaoxing	55.9	83.0	31.2	45.8	81.2	0.70
Ningbo	43.6	69.1	15.3	46.1	99.8	0.94
Zhoushan	22.5	37.1	6.0	16.1	106.5	0.57
Tāizhou	39.4	62.1	3.5	14.4	100.2	0.77
**PRD**						
Foshan	41.2	60.9	17.6	44.1	85.5	0.89
Guangzhou	41.2	64.6	11.2	53.0	81.8	0.97
Huizhou	24.0	50.0	8.8	17.3	91.5	0.77
Jiangmen	34.8	53.0	17.8	33.3	71.8	0.89
Zhongshan	31.2	45.4	10.3	24.2	71.2	1.13
Dongguan	38.7	51.6	15.0	34.0	113.5	0.80
Shenzhen	28.7	49.1	6.5	35.7	71.9	0.92
Zhuhai	29.7	54.0	5.9	27.6	84.8	0.81
**Cheng-Yu**						

Chengdu	69.5	116.6	14.0	63.1	99.4	1.20
Ziyang	32.5	75.1	37.2	11.3	108.3	0.74
Suining	49.1	86.9	10.4	20.0	84.9	0.85
Chongqing	55.4	85.1	13.8	52.4	58.5	1.22
Meishan	66.9	97.5	13.9	29.5	102.8	0.59
Neijiang	61.5	80.6	25.5	26.8	97.3	0.60

Table S5. Difference between measured and estimated concentrations in 2015

**City**	$\mathbf{D_{PM_{2.5}}}$	$\mathbf{D_{PM_{10}}}$	$\mathbf{D_{SO_2}}$	$\mathbf{D_{NO_2}}$	$\mathbf{D_{O_3}}$	$\mathbf{D_{CO}}$
**Jing-Jin-Ji**						
Zhangjiakou	14.7	13.1	-0.6	6.6	-0.6	0.21
Chengde	7.1	6.2	7.2	2.3	-10.5	0.21
Beijing	-5.1	11.4	11.1	-2.8	-3.0	0.07
Tangshan	-12.2	-19.6	-11.7	-9.0	-4.4	-0.42
Qinhuangdao	3.8	0.9	-0.6	-4.0	10.6	0.07
Baoding	-18.5	-28.8	-9.1	-7.0	-3.5	-0.26
Langfang	-1.6	-4.1	5.2	-0.2	2.4	0.01
Tianjin	1.2	-1.4	0.2	2.3	9.3	0.02
Shijiazhuang	-4.6	-5.0	4.0	-2.7	2.9	0.13
Hengshui	-2.9	-9.5	6.6	2.8	-7.8	0.07
Cangzhou	9.9	14.1	0.3	1.9	0.1	0.24
Xingtai	-5.5	-9.5	-6.2	-8.4	5.8	-0.10
Handan	-4.4	-14.6	2.6	-1.6	2.7	0.09
**YRD**						
Yangzhou	1.6	-4.0	-2.1	1.3	-6.6	0.03
Tàizhou	-1.9	-6.5	0.1	3.9	1.6	-0.01
Nantong	-0.8	-0.3	-4.5	1.0	-1.8	0.03
Nanjing	0.3	-3.3	2.2	-7.4	-8.7	0.04
Zhenjiang	-0.7	8.5	-0.2	-1.4	-2.8	0.00
Changzhou	-0.9	-5.6	-3.0	-2.6	1.5	-0.04
Wuxi	-2.5	-3.2	-1.4	-0.6	-2.4	-0.04
Suzhou	-0.9	0.0	0.8	-5.6	4.3	-0.02
Shanghai	1.1	4.2	3.4	-0.8	-0.1	-0.01
Huzhou	-0.2	3.5	2.0	1.4	-6.7	0.01
Jiaxing	0.4	0.2	-1.0	1.1	-5.4	0.05
Hangzhou	-3.2	-6.3	2.3	-5.6	-2.7	-0.05
Shaoxing	-1.4	-2.5	-4.2	-0.9	6.0	0.06
Ningbo	1.1	0.5	0.2	-3.4	-0.5	-0.06
Zhoushan	6.9	10.0	2.0	5.6	-2.8	0.09
Tāizhou	2.6	2.6	4.9	8.5	-3.2	0.02
**PRD**						
Foshan	-1.9	-2.0	-0.6	-3.9	-3.9	0.04
Guangzhou	-2.5	-4.1	1.2	-7.6	0.9	-0.01

Huizhou	3.1	0.3	1.1	3.9	-1.0	0.04
Jiangmen	-0.3	0.0	-1.8	-1.8	1.3	0.04
Zhongshan	1.5	3.7	1.1	4.1	2.8	-0.07
Dongguan	-2.3	0.6	-1.6	-0.3	-9.7	0.03
Shenzhen	1.0	0.7	1.7	-2.8	8.4	-0.03
Zhuhai	1.3	-1.0	2.6	0.8	-3.7	0.06
**Cheng-Yu**						
Chengdu	-7.1	-11.5	1.3	-12.4	-4.8	-0.10
Ziyang	8.3	5.1	-7.0	7.7	-6.9	0.06
Suining	0.4	-1.2	2.4	3.3	-0.9	0.03
Chongqing	-0.4	0.1	2.2	-8.6	7.2	-0.10
Meishan	-4.0	-3.5	2.0	0.5	-7.2	0.12
Neijiang	-1.0	3.5	-1.7	1.3	-5.5	0.08

Table S6. Overestimate or Underestimate Percentage ($P^{O/U}$) of self-made pollution severity in 2015

City	$P_{PM_{2.5}}$	$P_{PM_{10}}$	P_{SO_2}	P_{NO_2}	P_{O_3}	P_{CO}	Average $P^{O/U}$
**Jing-Jin-Ji**							
Zhangjiakou	79.1%	19.8%	-1.8%	34.7%	-0.6%	33.4%	27.4%
Chengde	20.6%	7.7%	50.5%	7.4%	-9.4%	26.6%	17.2%
Beijing	-6.0%	12.8%	627.2%	-5.4%	-3.0%	5.7%	105.2%
Tangshan	-12.7%	-12.2%	-19.4%	-13.0%	-4.5%	-16.4%	-13.0%
Qinhuangdao	8.8%	0.9%	-1.6%	-8.2%	19.5%	4.9%	4.1%
Baoding	-14.9%	-14.1%	-14.2%	-11.5%	-3.4%	-12.1%	-11.7%
Langfang	-1.9%	-2.9%	28.4%	-0.4%	2.8%	1.0%	4.5%
Tianjin	1.7%	-1.2%	0.7%	5.9%	13.7%	1.8%	3.8%
Shijiazhuang	-4.9%	-3.3%	9.0%	-5.2%	3.8%	10.4%	1.6%
Hengshui	-2.8%	-5.2%	22.4%	6.8%	-6.7%	5.0%	3.3%
Cangzhou	16.6%	13.1%	0.9%	4.7%	0.1%	24.6%	10.0%
Xingtai	-5.2%	-5.2%	-9.4%	-12.4%	8.0%	-5.4%	-4.9%
Handan	-4.7%	-8.0%	6.1%	-3.4%	3.5%	5.7%	-0.1%
**YRD**							
Yangzhou	3.1%	-3.8%	-7.9%	4.6%	-5.8%	3.2%	-1.1%
Tàizhou	-3.1%	-5.9%	0.2%	14.8%	1.7%	-1.5%	1.0%
Nantong	-1.4%	-0.3%	-13.4%	2.7%	-1.7%	3.4%	-1.8%
Nanjing	0.6%	-3.3%	12.7%	-12.9%	-7.9%	4.2%	-1.1%
Zhenjiang	-1.2%	11.3%	-0.8%	-3.3%	-2.5%	0.0%	0.6%
Changzhou	-1.4%	-5.2%	-9.1%	-5.6%	1.6%	-3.8%	-3.9%
Wuxi	-3.9%	-3.3%	-5.0%	-1.5%	-2.4%	-3.4%	-3.2%
Suzhou	-1.6%	0.0%	3.9%	-9.7%	4.6%	-2.2%	-0.8%
Shanghai	2.1%	6.1%	25.5%	-1.8%	-0.1%	-1.1%	5.1%
Huzhou	-0.3%	4.9%	13.5%	3.5%	-5.8%	0.7%	2.7%
Jiaxing	0.8%	0.2%	-4.5%	2.6%	-4.7%	7.6%	0.3%
Hangzhou	-5.5%	-7.0%	17.2%	-10.7%	-2.8%	-5.2%	-2.3%

Shaoxing	-2.5%	-3.1%	-13.5%	-2.1%	7.3%	7.9%	-1.0%
Ningbo	2.5%	0.7%	1.4%	-7.5%	-0.5%	-5.9%	-1.5%
Zhoushan	30.9%	26.9%	32.9%	34.8%	-2.6%	16.2%	23.2%
Tāizhou	6.5%	4.1%	140.7%	59.1%	-3.2%	2.5%	35.0%
**PRD**							
Foshan	-4.6%	-3.3%	-3.3%	-8.9%	-4.6%	4.4%	-3.4%
Guangzhou	-6.0%	-6.3%	10.5%	-14.3%	1.1%	-0.8%	-2.6%
Huizhou	13.1%	0.5%	12.8%	22.5%	-1.1%	5.5%	8.9%
Jiangmen	-0.9%	0.0%	-10.3%	-5.4%	1.8%	4.1%	-1.8%
Zhongshan	5.0%	8.1%	10.2%	16.8%	3.9%	-6.0%	6.3%
Dongguan	-5.9%	1.2%	-10.8%	-0.8%	-8.5%	3.7%	-3.5%
Shenzhen	3.6%	1.5%	26.9%	-7.7%	11.7%	-3.4%	5.4%
Zhuhai	4.5%	-1.8%	43.4%	2.7%	-4.4%	7.5%	8.6%
**Cheng-Yu**							
Chengdu	-10.2%	-9.9%	9.6%	-19.6%	-4.8%	-8.5%	-7.2%
Ziyang	25.5%	6.8%	-18.8%	67.7%	-6.4%	7.9%	13.8%
Suining	0.7%	-1.4%	22.8%	16.3%	-1.1%	3.5%	6.8%
Chongqing	-0.7%	0.1%	15.8%	-16.5%	12.4%	-8.5%	0.4%
Meishan	-6.0%	-3.6%	14.5%	1.7%	-7.0%	19.9%	3.3%
Neijiang	-1.6%	4.4%	-6.8%	4.8%	-5.6%	13.8%	1.5%

-The positive values are overestimates and the negative ones are underestimates.
